# Transition-metal-free synthesis of arylboronates via thermal generation of aryl radicals from triarylbismuthines in air

**DOI:** 10.3762/bjoc.20.216

**Published:** 2024-10-11

**Authors:** Yuki Yamamoto, Yuki Konakazawa, Kohsuke Fujiwara, Akiya Ogawa

**Affiliations:** 1 Graduate Faculty of Interdisciplinary Research, University of Yamanashi, 4-4-37 Takeda, Kofu, Yamanashi 400-8510, Japan; 2 Department of Applied Chemistry, Graduate School of Engineering, Osaka Metropolitan University, 1-1 Gakuen-cho, Nakaku, Sakai, Osaka 599-8531, Japan; 3 Organization for Research Promotion, Osaka Metropolitan University, 1-1 Gakuen-cho, Nakaku, Sakai, Osaka 599-8531, Japan

**Keywords:** arylboronates, bis(pinacolato)diboron, radical reactions, transition-metal-free synthesis, triarylbismuthines

## Abstract

A simple and versatile synthesis of arylboronates has been achieved by using triarylbismuthines as aryl radical sources under transition-metal-free and open-air conditions. Conventional methods required photoirradiation or electrolysis to generate aryl radicals from triarylbismuthines. In this study, it was found that simply heating the solution of triarylbismuthines in benzotrifluoride (BTF) in air successfully led to the generation of aryl radicals, and the subsequent reaction with bis(pinacolato)diboron afforded a variety of arylboronates in moderate to good yields.

## Introduction

Arylboronates are one of the fundamental aryl compounds in organic synthesis, especially in cross-coupling reactions [1–9], and their applications are widespread, including dye synthesis, pharmaceutical and agrochemical synthesis, and industrial manufacturing [10–11]. In recent years, a variety of transition-metal-catalyzed reactions and photoredox reactions using arylboronates as aryl sources have been energetically investigated for the construction of carbon–carbon or carbon–heteroatom bonds [12–15].

The preparation of arylboronates often requires pre-functionalized substrates with halogen or triflate groups. Recently, transition-metal-catalyzed direct borylation of arenes via C–H bond activation has been reported, although the design of the substrate and ligands is somewhat complicated [16–22]. Since the complete removal of catalyst-derived metal residues from the final products is generally difficult, there is concern about side effects or adverse effects on functional expression when used in pharmaceutical and material synthesis. In addition, many transition metal catalysts are very expensive, unstable, and difficult to handle. For these reasons, the development of new synthetic methods of arylboronates using stable and versatile reagents under transition-metal-free conditions has recently attracted much attention [23–27]. In particular, the use of radical reactions has been considered as one of the effective methods, since diborons can capture the in situ-generated carbon-centered radicals [28–36].

**Scheme 1:**
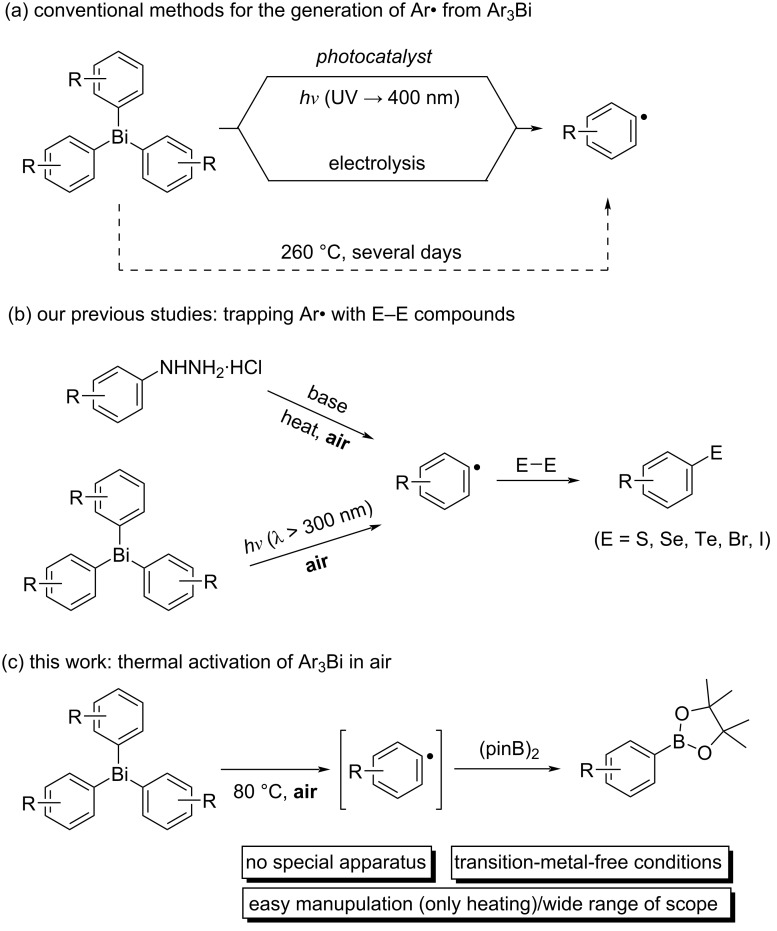
(a) Conventional methods for the generation of Ar^•^ from Ar_3_Bi, (b) our previous studies, and (c) this work.

Among the aryl sources in organic synthesis, triarylbismuthines are shelf-stable and easy-to-handle reagents with appropriate reactivities in transition-metal-catalyzed reactions and radical reactions, and their derivatives can be easily synthesized by common Grignard reactions [37–44]. Three activation methods have been reported for their use as aryl radical sources. It has been reported that the homolysis of Ar–Bi bonds could be achieved by photoirradiation in the presence of photocatalysts or UV light irradiation without metal catalysts [45–48]. Similar homolysis by electrolysis has also been reported [49]. These two activation methods required special equipment (i.e., light sources or electronic devices). To achieve thermal homolysis of the Ar–Bi bonds, the reaction conditions were harsh, requiring heating at 260 °C for several days [50]. Thus, only two examples of the use of triarylbismuthines as aryl radical sources have been reported for the synthesis of arylboronates, which proceeds under light irradiation conditions (Scheme 1a) [47–48].

Our group has investigated various transition-metal-free methods for the generation of aryl radicals from shelf-stable aryl compounds (Scheme 1b). For example, the heating of arylhydrazine hydrochlorides (ArNHNH_2_·HCl) in the presence of base under open-air conditions successfully led to the generation of aryl radicals and the subsequent trapping with E–E compounds (E = S, Se, Te, Br, and I) successfully formed new C–E bonds (Scheme 1b) [51–58]. We also demonstrated that the photoirradiation (λ > 300 nm) of the triarylbismuthines in air successfully allowed the generation of the corresponding aryl radicals without photocatalysts, and the trapping with diselenides afforded a variety of diaryl selenides [59]. Based on these backgrounds of our studies and the fundamental property, i.e., the weak bond dissociation energy of the Ph–Bi bond (46 kcal/mol) [60], we hypothesized that aryl radicals generated from triarylbismuthines by our developed methods would be successfully trapped by diboron to form a new C–B bond.

In this study, we report a facile and versatile synthesis of arylboronates using triarylbismuthines as aryl radical sources under transition-metal-free and open-air conditions (Scheme 1c). This method could be carried out without any special apparatus, and the mild conditions led to the wide range of applications.

## Results and Discussion

Initially, we used triphenylbismuthine (**1a**) and bis(pinacolato)diboron (**2**) as the model substrates to optimize the reaction conditions (Table 1). We first investigated the solubility of **1** and **2** in various solvents. It was found that both **1** and **2** showed excellent solubility towards benzotrifluoride (BTF) [61] (for diboron **2**: 637 mg/mL (BTF); 567 mg/mL (AcOEt)). Therefore, BTF is chosen as the solvent.

**Table 1:** Optimization of the reaction conditions for synthesis of **3a** from BiPh_3_ (**1a**) and (pinB)_2_ (**2**).

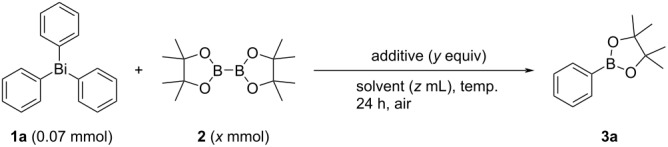						

Entry	**2** (mmol)	Additive (equiv)	Solvent (mL)	Temp (°C)	Yield **3a** (%)^a^	Recovery **1a** (%)^a^

**1**	**2.0**	**–**	**BTF (0.4)**	**80**	**68 (53)**	**trace**
2	0.5	NaOMe (1.2)	BTF (0.2)	80	N. D.	62
3	3.0	–	BTF (0.4)	80	62	trace
4	0.5	–	BTF (0.4)	80	36	30
5^b^	2.0	–	BTF (0.4)	80	58	trace
6	2.0	–	BTF (0.4)	60	59	trace
7	2.0	–	BTF (0.4)	100	61	trace
8	2.0	(PhS)_2_ (0.01)	BTF (0.4)	80	63	trace
9	2.0	–	CHCl_3_ (0.4)	80	36	17
10	2.0	–	CH_3_CN (0.4)	80	51	17
11	2.0	–	DMF (0.4)	80	38	48
12	2.0	–	DMSO (0.4)	80	18	80
13	2.0	–	EtOH (0.4)	80	45	16

^a^Yields were determined by ^1^H NMR spectroscopy based on **1a** as three transferable aryl groups (internal standard: 1,3,5-trioxane). Isolated yield was shown in parentheses. ^b^Under O_2_ (0.1 MPa).

Surprisingly, heating the mixture of **1** and **2** in BTF (0.4 mL) at 80 °C in air successfully afforded phenylboronic acid pinacol ester **3a** in 68% yield (Table 1, entry 1). In the presence of NaOMe as a base, the reaction did not proceed (Table 1, entry 2). Increasing or decreasing the amount of diboron **2** did not improve the yield of **3a** (Table 1, entry 2 vs entries 3 and 4). Under atmospheric oxygen, the yield of **3a** decreased slightly (Table 1, entry 5). The reaction was investigated at 60 °C and 100 °C, and it was found that the reaction was most efficient at 80 °C (Table 1, entry 2 vs entries 6 and 7). We have previously succeeded in generating a boron radical (pinB^•^) by photoirradiation of (Bpin)_2_ and found that the addition of (PhS)_2_ was effective in generating the boron radical [30]. We therefore investigated this reaction by adding (PhS)_2_ as a Lewis base, but the yield of **3a** was not improved and (PhS)_2_ was recovered almost quantitatively (Table 1, entry 8). Furthermore, instead of BTF, the reaction was carried out with similarly polar CHCl_3_, polar and aprotic CH_3_CN, DMF, DMSO, and protic EtOH, and it was found that BTF was the optimal solvent for the synthesis of **3a** (Table 1, entry 2 vs entries 9–13).

**Scheme 2:**
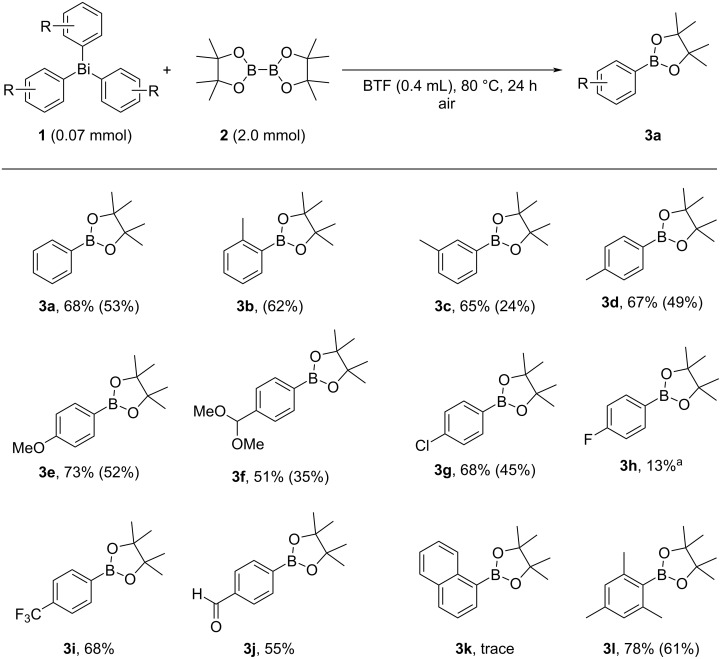
Scope for transition-metal-free synthesis of arylboronates **3** using triaylbismuthines **1** and diboron **2**. Yields were determined by ^1^H NMR spectroscopy based on **1** as three transferable aryl groups (internal standard: 1,3,5-trioxane). Isolated yield was shown in parentheses. ^a^CHCl_3_ (0.4 mL) was used as the solvent.

Based on the optimized conditions (entry 2 in Table 1), we next investigated the scope and limitations of the transition-metal-free synthesis of arylboronates **3** using functionalized triarylbismuthines **1** (Scheme 2). As shown in Scheme 2, a variety of triarylbismuthines could be used for the transition-metal-free synthesis of arylboronates. For example, the use of triarylbismuthines with *o*-methyl, *m*-methyl, *p*-methyl, *p*-methoxy, and *p*-chloro groups successfully afforded the corresponding arylboronates **3a**–**e** and **3g** in 62–73% yields, respectively. The low solubility of tri(*p*-fluorophenyl) and tri(1-naphthyl)bismuthines **1h** and **1k** in BTF resulted in the low conversion. This system could be applied to the unstable dimethyl acetal-substituted triphenylbismuthine **1f**, and **3f** was obtained in 51% yield. Interestingly, the use of triarylbismuthines **1i** and **1j** with strong electron-withdrawing groups such as trifluoromethyl and formyl groups was also tolerable, and the corresponding products **3i** and **3j** were selectively obtained in moderate yields. Notably, the bulky 2,4,6-trimethylphenyl group of bisumuthine **1l** did not inhibit the transformation, and the boronate **3l** was obtained in 78% yield. The isolation of arylboronates **3c**, **3i**, and **3j** was somewhat difficult due to strong adsorption or decomposition on silica gel. Since some arylboronates are somewhat unstable, it is desirable to synthesize such compounds and then use them in a one-pot manner for the following reactions without isolation.

**Scheme 3:**
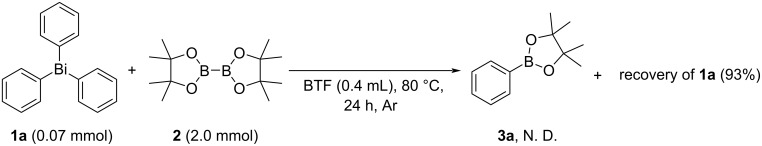
Control experiment of the metal-free borylation under an argon atmosphere.

To gain insight into the reaction pathways, several control experiments were investigated. When the reaction was carried out in an argon atmosphere using the strict Schlenk technique, the desired product **3a** was not obtained at all and 93% of **1a** was recovered (Scheme 3).

**Figure 1:**
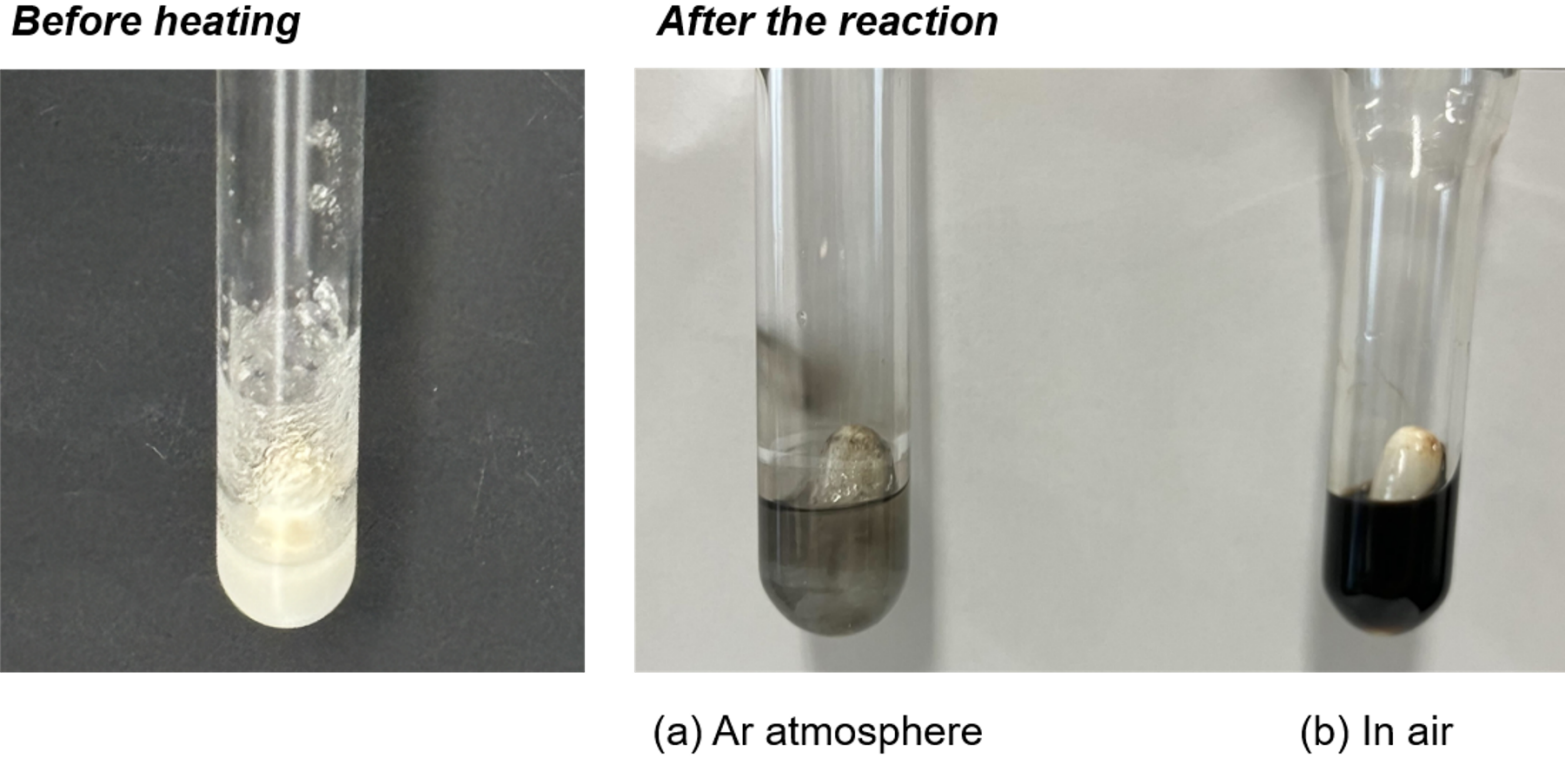
Comparison of the crude mixture of the reactions under (a) argon atmosphere or (b) open-air.

Figure 1 shows the comparison of the crude mixture of the reactions under argon atmosphere and in the open-air. In the absence of oxygen, the color of the reaction mixture changed only slightly. In contrast, the reaction in air resulted in the formation of black and a small amount of white insoluble solid (probably metallic bismuth or bismuth oxide) [55], and **3a** was successfully obtained with almost complete consumption of **1a**. The results clearly indicate that air can play an important role in the thermal activation of triarylbismuthines to generate aryl radicals.

**Scheme 4:**
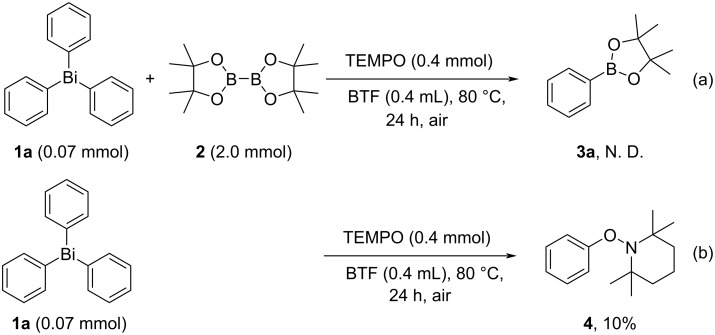
Radical-trapping experiments using TEMPO as a radical scavenger.

Furthermore, the yield of **3a** was dramatically reduced in the presence of 2,2,6,6-tetramethylpiperidine 1-oxyl free radical (TEMPO) as a radical scavenger, strongly suggesting that a radical pathway is involved in the key step of the arylboronate synthesis (Scheme 4a). In addition, the reaction of triphenylbismuthine **1a** (0.07 mmol) and TEMPO (0.4 mmol) under air resulted in the formation of **4** in 10% yield, which was confirmed and characterized by the ^1^H NMR measurement of the crude reaction mixture (Scheme 4b) [48]. These results clearly showed that the thermal generation of aryl radicals from triarylbismuthines is one of the key factors for the transition-metal-free synthesis of arylboronates, and oxygen (air) would play a role as a radical initiator for the thermal activation of triarylbismuthines.

In this system, an excess amount of diboron **2** was required for the reaction to proceed efficiently. To clarify the transformation of diboron **2** under the reaction conditions, the crude reaction mixture (entry 2 in Table 1) was analyzed by ^11^B NMR measurement. It was noteworthy that diboron **2** was reactive with air under the reaction conditions, and the decomposition of **2** to form pinB–O–Bpin and pinB–OH was confirmed. The decomposition was also occurred by heating the solution of diboron **2** in BTF in air; however, in the presence of TEMPO, the decomposition of **2** was sightly occurred, and almost all of **2** was recovered (see Supporting Information File 1). Based on the results, diboron **2** could also be activated via the thermal homolysis of the B–B bond in the presence of oxygen (air).

**Scheme 5:**
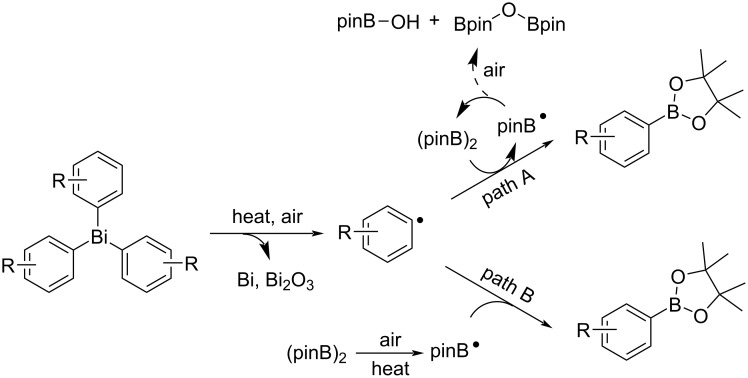
A proposed reaction pathway for the synthesis of arylboronates.

Based on the results of the control experiments and our previous studies, a proposed reaction pathway is shown in Scheme 5. First, thermal activation of triarylbismuthines in air forms aryl radicals together with the bismuth residues (i.e., metal bismuth and bismuth oxide). Alternatively, oxygen in air and/or boron-centered radicals thermally generated from (pinB)_2_ in air would react with triarylbismuthines to form the aryl radicals. The generated aryl radicals were then captured with (pinB)_2_ and the corresponding arylboronates were formed. Recombination of simultaneously formed pinB^•^ could regenerate (pinB)_2_, and some of the pinB^•^ would react with air to form pinB–O–Bpin and pinB–OH (path A). The corresponding arylboronates could also be formed via aryl radical trapping with pinB^•^ generated by heating (pinB)_2_ in air (path B).

## Conclusion

In this study, we have developed a novel method for the transition-metal-free synthesis of arylboronates using triarylbismuthines. Most of the previous methods to generate aryl radical species from triarylbismuthines required a special apparatus. In contrast, our method was very simple, and the corresponding aryl radicals were easily accessible by simply heating the solution of triarylbismuthines in air under mild conditions. Therefore, many triarylbismuthines could be used to form a variety of useful arylboronates in moderate to good yields with excellent product selectivity. We hope that this new approach to the generation of aryl radicals from triarylbismuthines will lead to an increased use of organobismuth compounds in synthetic organic chemistry. Further applications of organobismuth compounds as aryl radical precursors are currently under investigation.

## Experimental

**General comments:** Unless otherwise stated, all starting materials were purchased from commercial sources and used without further purification. All solvents were used without distillation. Triarylbismuthines **1** were synthesized according to the previously reported procedures [62]. ^1^H, ^13^C{^1^H}, and ^11^B NMR spectra were recorded in CDCl_3_ using a Bruker AVANCE III HD 500 spectrometer at 500, 126, and 160 MHz, respectively. ^1^H chemical shifts are reported in ppm relative to Me_4_Si using the solvent residual as the internal standard (δ = 7.26 ppm for chloroform). ^13^C chemical shifts are reported in ppm relative to Me_4_Si, referenced to the resonances of CDCl_3_ (δ = 77.2 ppm). ^11^B chemical shifts are reported in ppm recorded in CDCl_3_ using BF_3_·Et_2_O (δ = 0.0 ppm) as the internal standard.

**General procedure for transition-metal-free synthesis of arylboronates with triarylbismuthines 1 and diboron 2 (**Scheme 2**):** To a 10 mL two-neck flask were added triarylbismuthine **1** (0.07 mmol), bis(pinacolato)diboron **2** (2.0 mmol), and benzotrifluoride (BTF, 0.4 mL). The mixture was heated at 80 °C for 24 h in air. After the reaction was completed, the mixture was filtered through a short Celite pad with AcOEt (20 mL). The filtrate was concentrated under reduced pressure. Finally, the residue was purified by preparative thin-layer chromatography (eluent: AcOEt/hexane) to give the pure product **3**. Assuming that three aryl radicals are formed from triarylbismuthine **1**, the yield of **3** was determined from the weight of the isolated product based on three times the moles of triarylbismuthine **1**. Further details of the experimental procedures and characterization data are provided in Supporting Information File 1.

## Supporting Information

File 1 Investigation of the boron residue in the crude mixture by ^11^B NMR measurement, characterization data of the compounds, and copies of ^1^H NMR and ^13^C{^1^H} NMR spectra.

## Data Availability

All data that supports the findings of this study is available in the published article and/or the supporting information to this article.
