# Supplementary material for: Transition-metal-free synthesis of arylboronates via thermal generation of aryl radicals from triarylbismuthines in air
Source: Beilstein J Org Chem. 2024 Oct 11;20:2577–84. doi: 10.3762/bjoc.20.216 (PMC11472659; doi:10.3762/bjoc.20.216)

BEILSTEIN JOURNAL OF ORGANIC CHEMISTRY

Supporting Information

for

Transition-metal-free synthesis of arylboronates via thermal generation of aryl radicals from triarylbismuthines in air

Yuki Yamamoto, Yuki Konakazawa, Kohsuke Fujiwara and Akiya Ogawa

Beilstein J. Org. Chem. **2024,** *20,* 2577–2584. doi:10.3762/bjoc.20.216

Investigation of the boron residue in the crude mixture by ^{11}B NMR measurement, characterization data of the compounds, and copies of ^{1}H NMR and ^{13}C{^{1}H} NMR spectra

License and Terms: This is a supporting information file under the terms of the Creative Commons Attribution License (https://creativecommons.org/licenses/by/4.0). Please note that the reuse, redistribution and reproduction in particular requires that the author(s) and source are credited and that individual graphics may be subject to special legal provisions.
The license is subject to the *Beilstein Journal of Organic Chemistry* terms and conditions: (https://www.beilstein-journals.org/bjoc/terms)

CONTENTS

	page
Investigation of the boron residue in the crude mixture by ^{11}B NMR measurement	S2
Characterization data of the arylboronates **3**	S3–S5
Copies of ^{1}H NMR and ^{13}C NMR spectra of the arylboronates **3**	S6–S13

Investigation of the boron residue in the crude mixture by ^{11}B NMR measurement

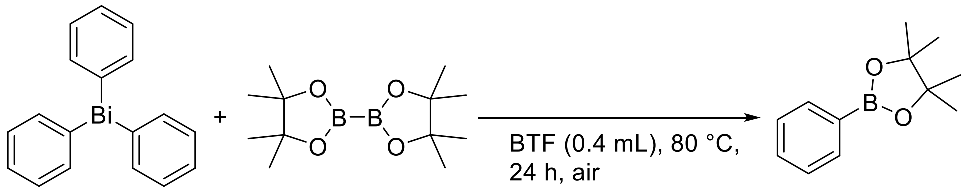

1a (0.07 mmol) **2** (2.0 mmol) **3a**, 68%

The reaction was carried out under the optimal condition (entry 2 in Scheme 2). The reaction mixture was then analyzed by ^{11}B NMR measurement in $CDCl_3$ using BF_3•Et_2O as the internal standard (δ= 0.0 ppm).

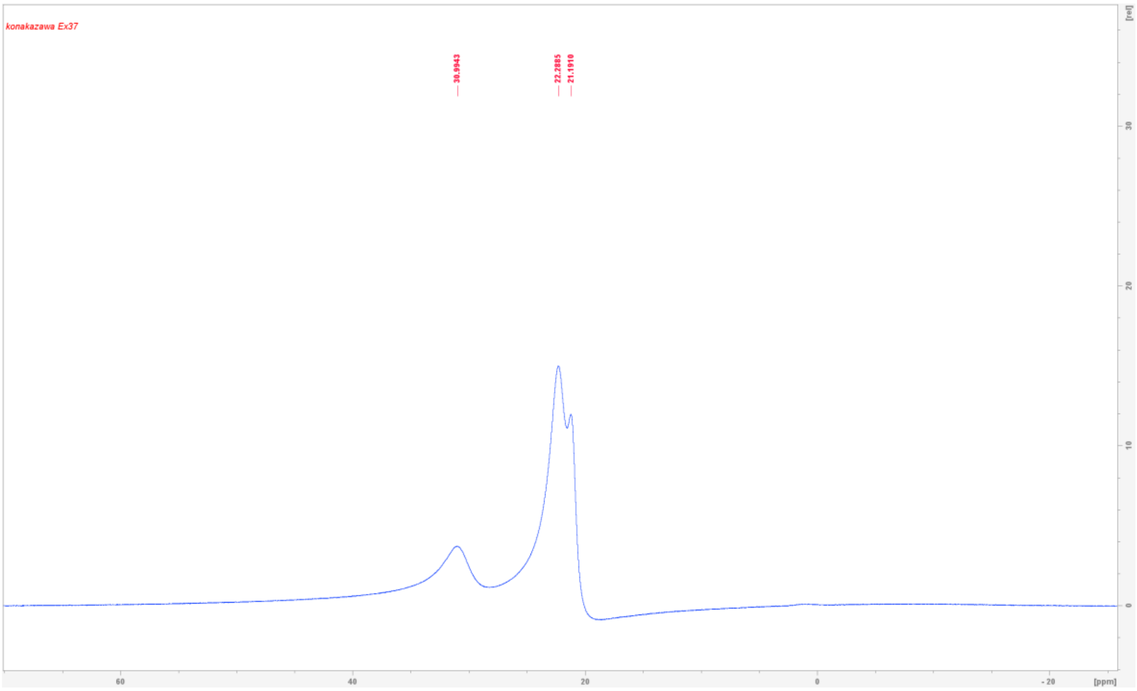

The peaks of bispinacolatodiboron **2** (δ_B = 30.5 ppm)[1] and the product **3a** (δ_B = 30.9 ppm)[2] were overlapped in the ^{11}B NMR spectra. In addition, two other peaks were observed that could be attributed as pinB–O–Bpin (δ_B = 21.2 ppm)[1] and pinB–OH (δ_B = 22.3 ppm)[3], respectively. Therefore, we estimated that some of **2** might be decomposed under the reaction conditions.

Characterization data of the arylboronates 3

4,4,5,5-Tetramethyl-2-phenyl-1,3,2-dioxaborolane (**3a**)[2]

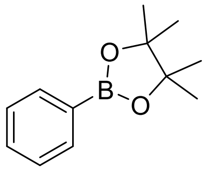

Light yellow solid, 22.6 mg, 53% yield; ^{1}H NMR (500 MHz, $CDCl_3$): δ 7.81 (d, *J* = 7.8 Hz, 2H), 7.46–7.44 (m, 1H), 7.38–7.35 (m, 2H), 1.35 (s, 12H); ^{13}C NMR (126 MHz, $CDCl_3$): δ 134.8, 131.3, 127.8, 83.8, 24.9. The ^{13}C signal bound to boron could not be observed due to quadrupolar relaxation.

4,4,5,5-Tetramethyl-2-(o-tolyl)-1,3,2-dioxaborolane (**3b**)[4]

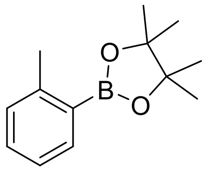

Colorless solid, 28.2 mg, 62% yield; ^{1}H NMR (500 MHz, $CDCl_3$): δ 7.77–7.75 (m, 1H), 7.33–7.30 (m, 1H), 7.17–7.14 (m, 2H), 2.54 (s, 3H), 1.34 (s, 12H) ^{13}C NMR (126 MHz, $CDCl_3$): δ 144.9, 135.9, 130.8, 129.8, 124.7, 83.4, 24.9, 22.2. The ^{13}C signal bound to boron could not be observed due to quadrupolar relaxation.

4,4,5,5-tetramethyl-2-(m-tolyl)-1,3,2-dioxaborolane (**3c**)[4]

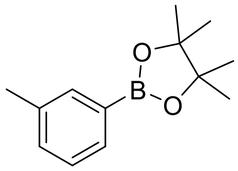

Colorless oil, 10.8 mg, 24% yield; 1H NMR (500 MHz, $CDCl_3$): δ 7.64 (s, 1H), 7.62–7.60 (m, 1H), 7.28–7.26 (m, 2H), 2.35 (s, 3H), 1.35 (s, 12H); ^{13}C NMR (126 MHz, $CDCl_3$): δ 137.2, 135.4, 132.1, 131.8, 127.7, 83.7, 24.9, 21.3. The ^{13}C signal bound to boron could not be observed due to quadrupolar relaxation.

4,4,5,5-Tetramethyl-2-(p-tolyl)-1,3,2-dioxaborolane (**3d**)[5]

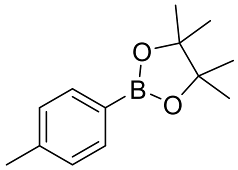

Light brown oil, 22.6 mg, 49% yield; ^{1}H NMR (500 MHz, $CDCl_3$): δ 7.70 (d, *J* = 7.9 Hz, 2H), 7.19 (d, *J* = 7.6 Hz, 2H), 2.37 (s, 3H), 1.34 (s, 12H); ^{13}C NMR (126 MHz, $CDCl_3$): δ 141.4, 134.8, 128.5, 83.6, 24.9, 21.8. The ^{13}C signal bound to boron could not be observed due to quadrupolar relaxation.

2-(4-Methoxyphenyl)-4,4,5,5-tetramethyl-1,3,2-dioxaborolane (**3e**)[4]

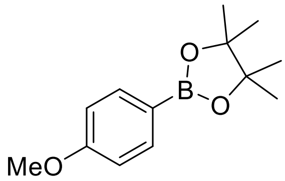

Colorless oil, 25.6 mg, 52% yield; ^{1}H NMR (500 MHz, $CDCl_3$): δ 7.75 (d, *J* = 8.7 Hz, 2H), 6.90 (d, *J* = 8.7 Hz, 2H), 3.82 (s, 3H), 1.33 (s, 12H); ^{13}C NMR (126 MHz, $CDCl_3$): δ 162.1, 136.5, 113.3, 83.6, 55.1, 24.9. The ^{13}C signal bound to boron could not be observed due to quadrupolar relaxation.

2-(4-(Dimethoxymethyl)phenyl)-4,4,5,5-tetramethyl-1,3,2-dioxaborolane (**3f**)[5]

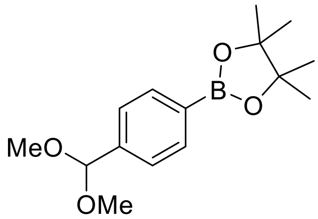

Colorless oil, 20.6 mg, 35% yield; ^{1}H NMR (500 MHz, $CDCl_3$): δ 7.82 (d, *J* = 8.1 Hz, 2H), 7.45 (d, *J* = 7.9 Hz, 2H), 5.42 (s, 1H), 3.31 (s, 6H), 1.35 (s, 12H); ^{13}C NMR (126 MHz, $CDCl_3$): δ 140.9, 134.7, 126.1, 102.9, 83.8, 52.6, 24.9. The ^{13}C signal bound to boron could not be observed due to quadrupolar relaxation.

2-(4-Chlorophenyl)-4,4,5,5-tetramethyl-1,3,2-dioxaborolane (**3g**)[4]

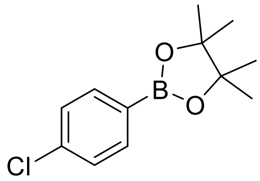

Colorless oil, 22.5 mg, 45% yield; ^{1}H NMR (500 MHz, $CDCl_3$): δ 7.73 (d, J = 8.3Hz, 2H), 7.34 (d, J = 8.4 Hz, 2H), 1.34 (s, 12H); ^{13}C NMR (126 MHz, $CDCl_3$): δ 137.5, 136.1, 128.1, 84.0, 24.9. The ^{13}C signal bound to boron could not be observed due to quadrupolar relaxation.

2-Mesityl-4,4,5,5-tetramethyl-1,3,2-dioxaborolane (**3l**)[2]

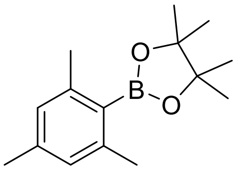

Colorless oil, 31.7 mg, 61% yield; ^{1}H NMR (500 MHz, $CDCl_3$): δ 6.77 (s, 2H), 2.36 (s, 6H), 2.24 (s, 3H), 1.37 (s, 12H); ^{13}C NMR (126 MHz, $CDCl_3$): δ 142.1, 138.9, 127.5, 83.5, 25.0, 22.2, 21.3. The ^{13}C signal bound to boron could not be observed due to quadrupolar relaxation.

The isolation of arylboronates **3c**, **3i**, and **3j** with sufficient isolated yields was somewhat difficult due to strong adsorption or decomposition on silica gel. The characterization of the arylboronates was determined from previously reported spectra.[2, 4, 6]

References

[1] De Smet, G.; Bai, X.; Mensch, C.; Sergeyev, S.; Evano, G.; Maes, B. U. W. *Angew. Chem. Int. Ed.* **2022**, *61*, e202201751.

[2] Tan, X.; Wang, X.; Hua Li, Z.; Wang, H. *J. Am. Chem. Soc.* **2022**, *144*, 23286–23291.

[3] Ji, P.; Sawano, T.; Lin, Z.; Urban, A.; Boures, D.; Lin, W. *J. Am. Chem. Soc.* **2016**, *138*, 14860–14863.

[4] Zhang, J.; Wei, R.; Ren, C.; Liu, L. L.; Wu, L. *J. Am. Chem. Soc.* **2023**, *145*, 15619–15629.

[5] Clary, J. W.; J. Rettenmaier, T. J.; Snelling, R.; Bryks, W.; Banwell, J.; Wipke, W. T.; Singaram, B. *J. Org. Chem.* **2011**, *76*, 9602–9610.

[6] Li, D.; Wei, L.; Xiong, W.; Jiang, H.; Qi, C. *J. Org. Chem.* **2023**, *88*, 5231–5237.

Copies of ^{1}H NMR and ^{13}C NMR spectra of the arylboronates 3

Figure S1. ^{1}H and $^{13}C\{^{1}H\}$ NMR spectra of compound **3a**

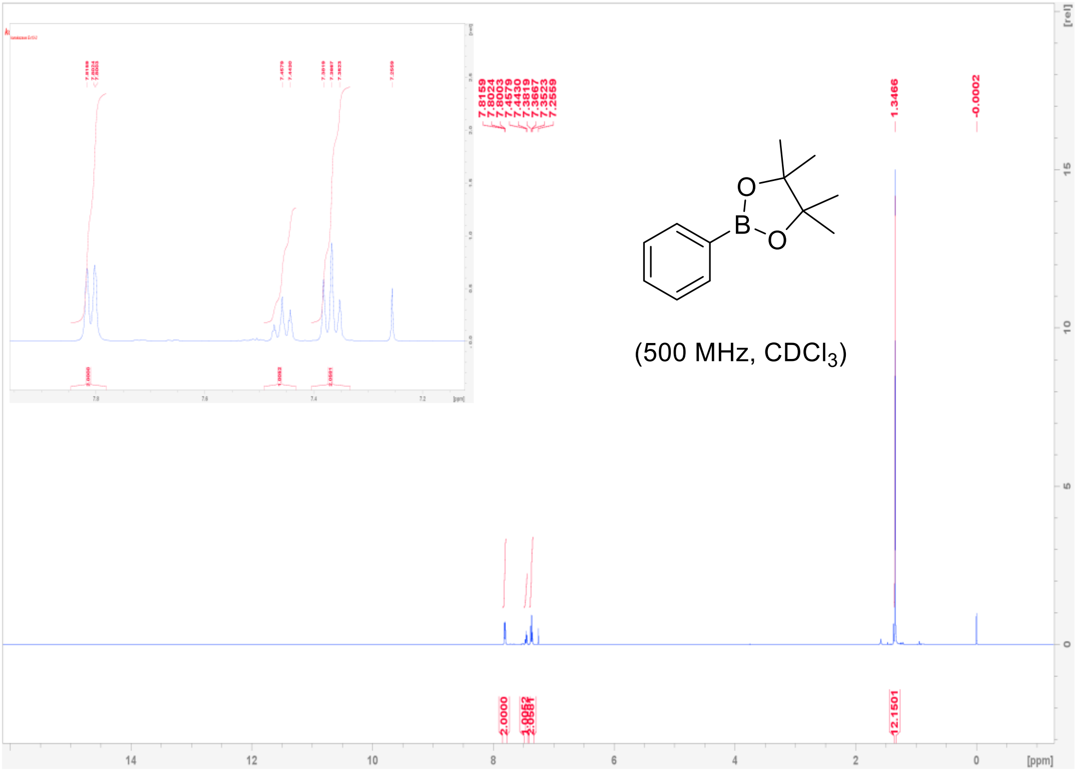

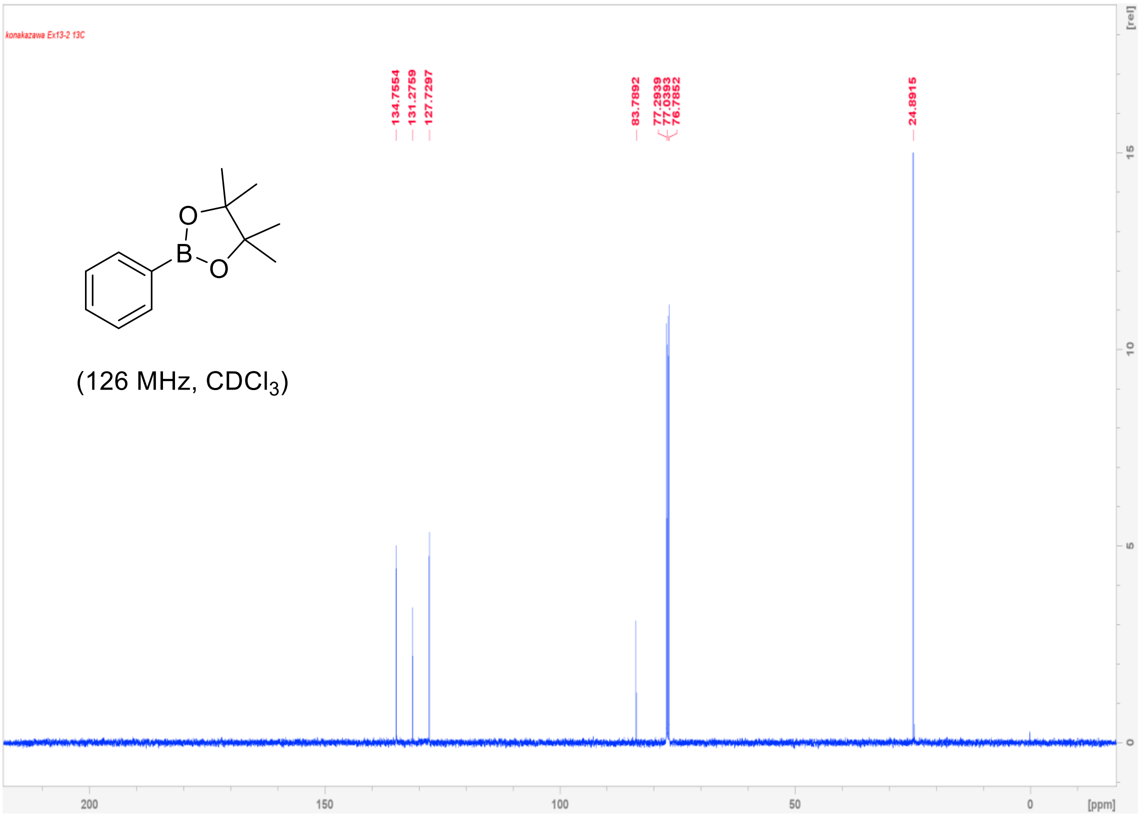

Figure S2. ^{1}H and ^{13}C{^{1}H} NMR spectra of compound **3b**

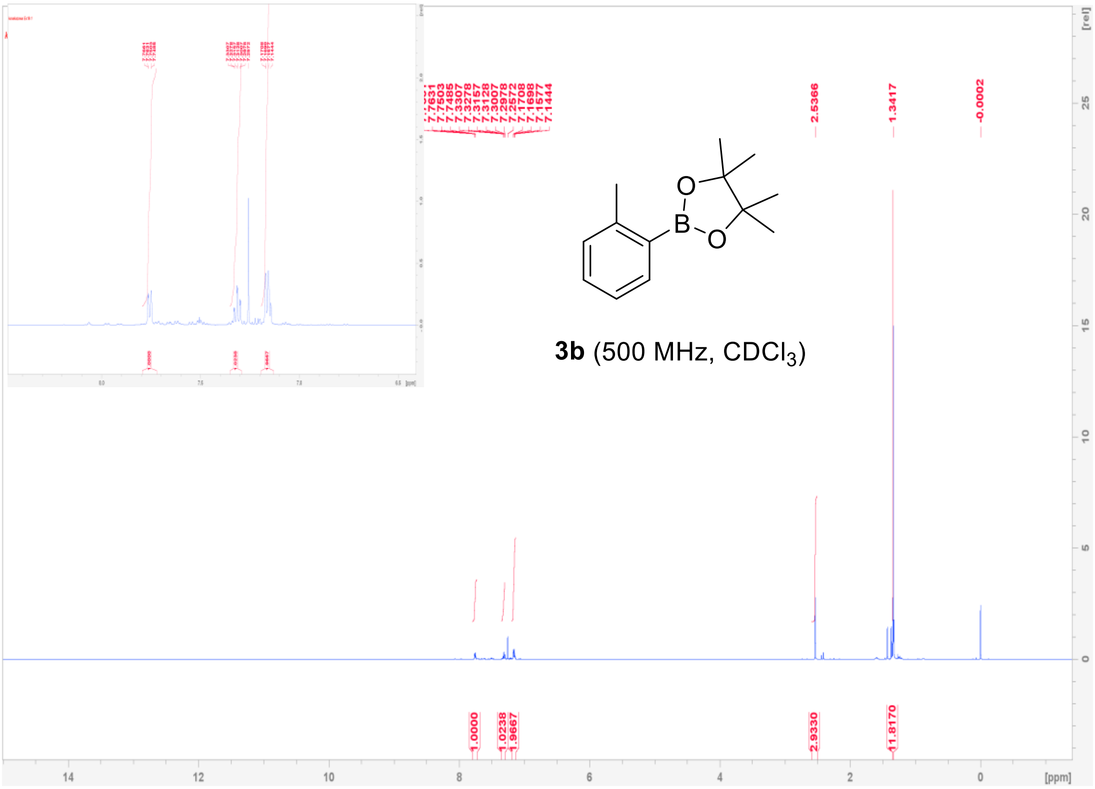

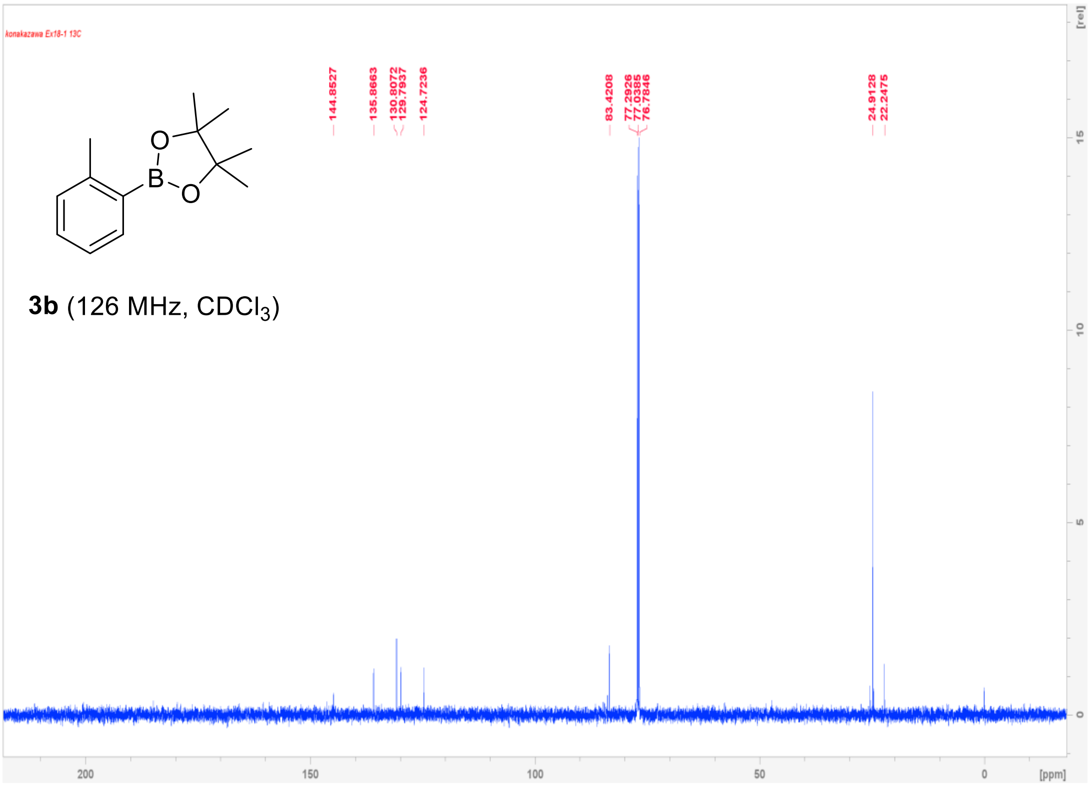

Figure S3. ^{1}H and $^{13}C\{^{1}H\}$ NMR spectra of compound **3c**

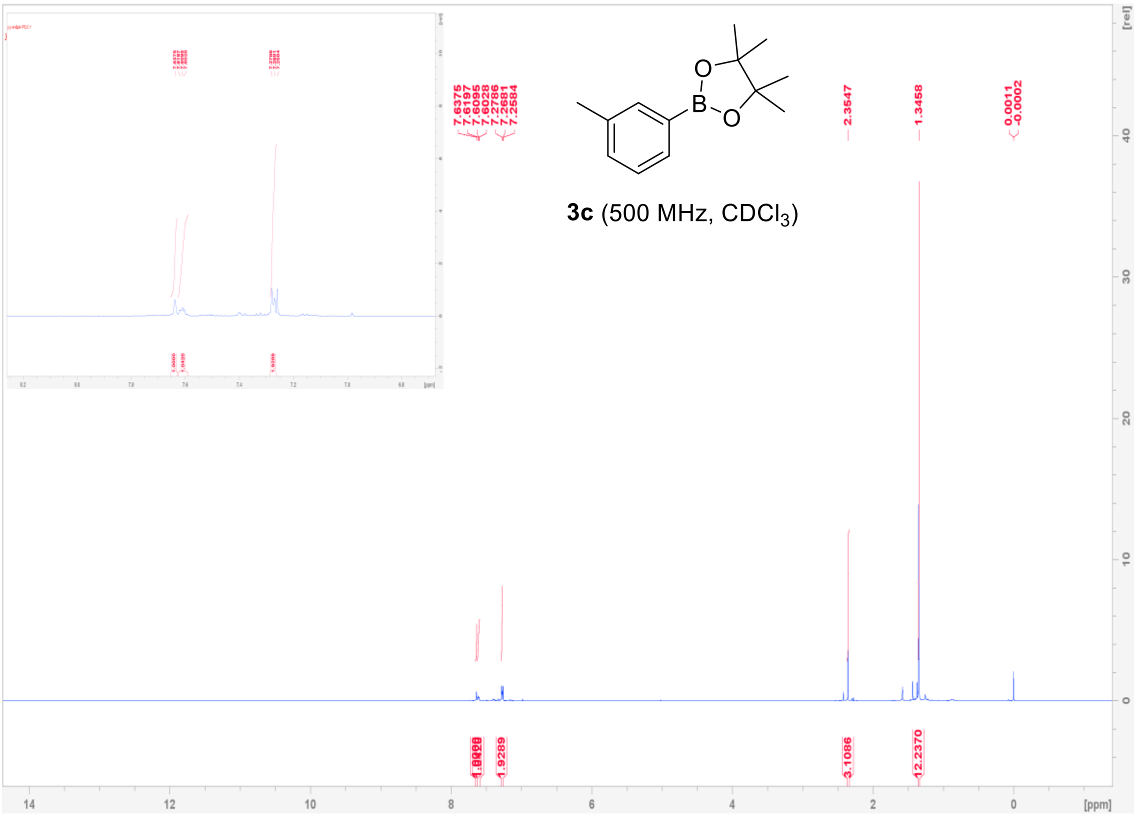

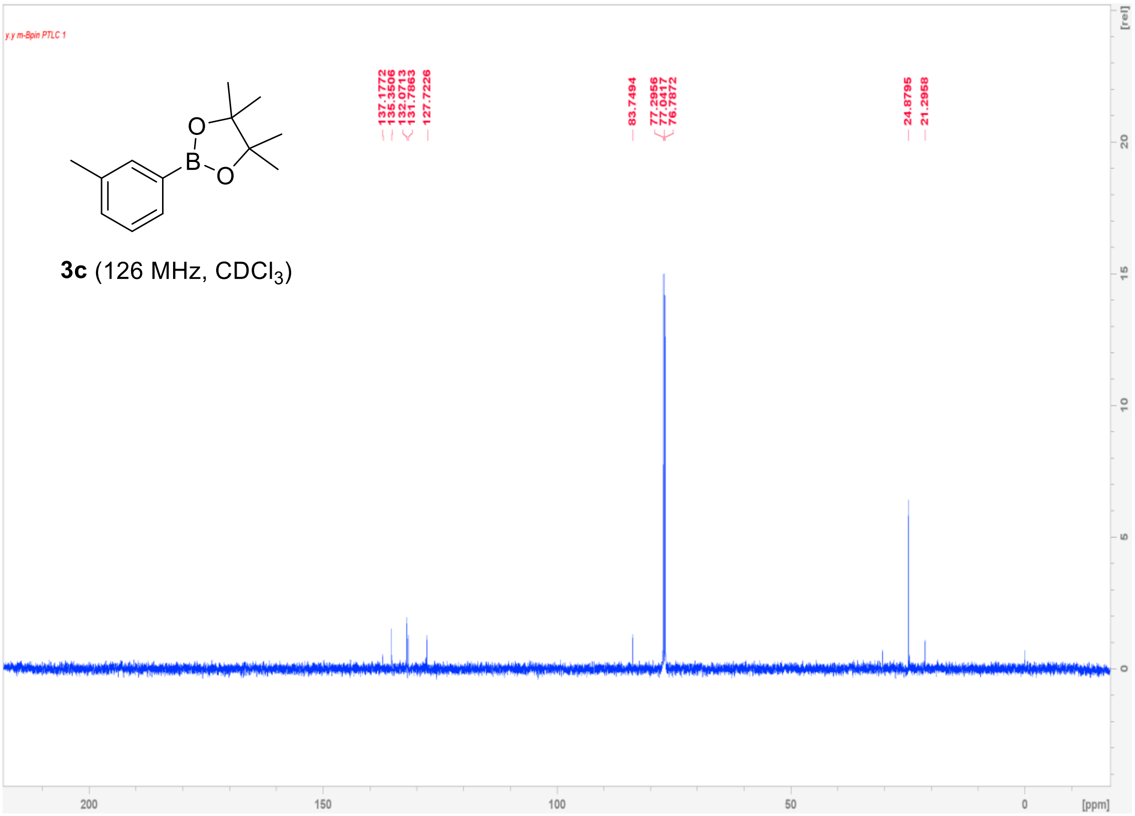

Figure S4. ^{1}H and $^{13}C\{^{1}H\}$ NMR spectra of compound **3d**

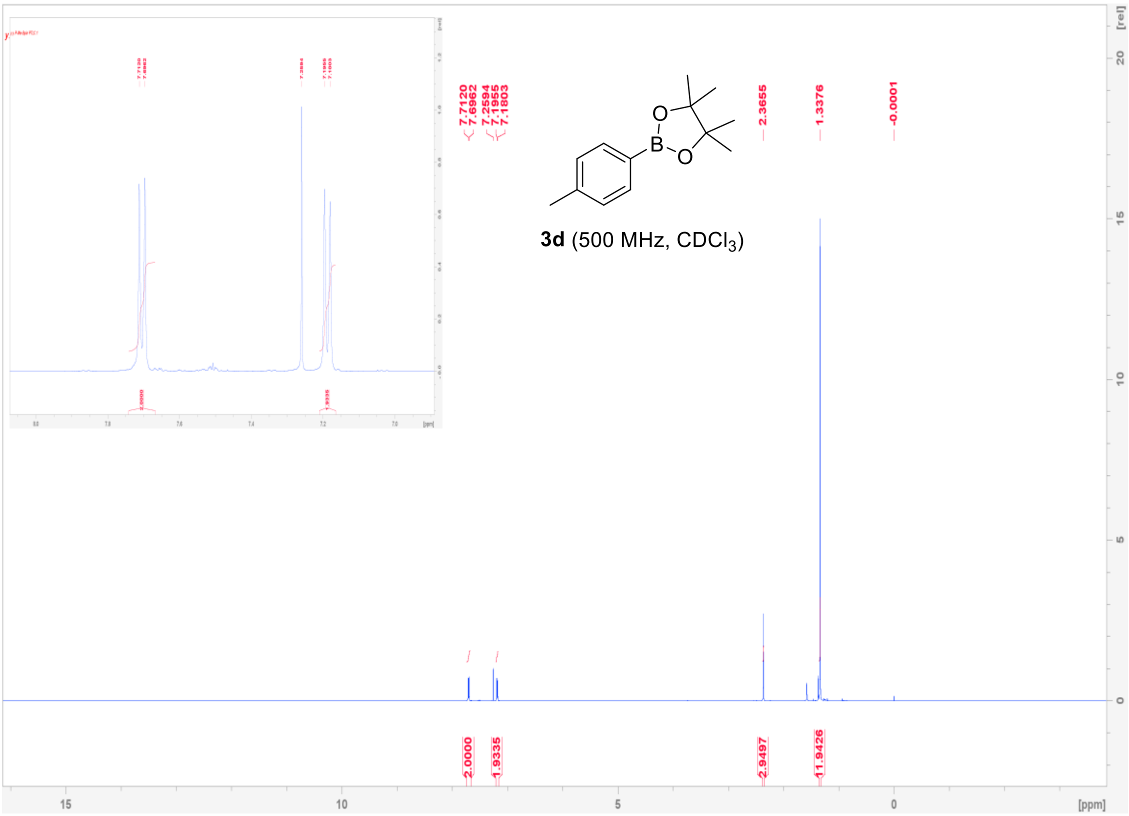

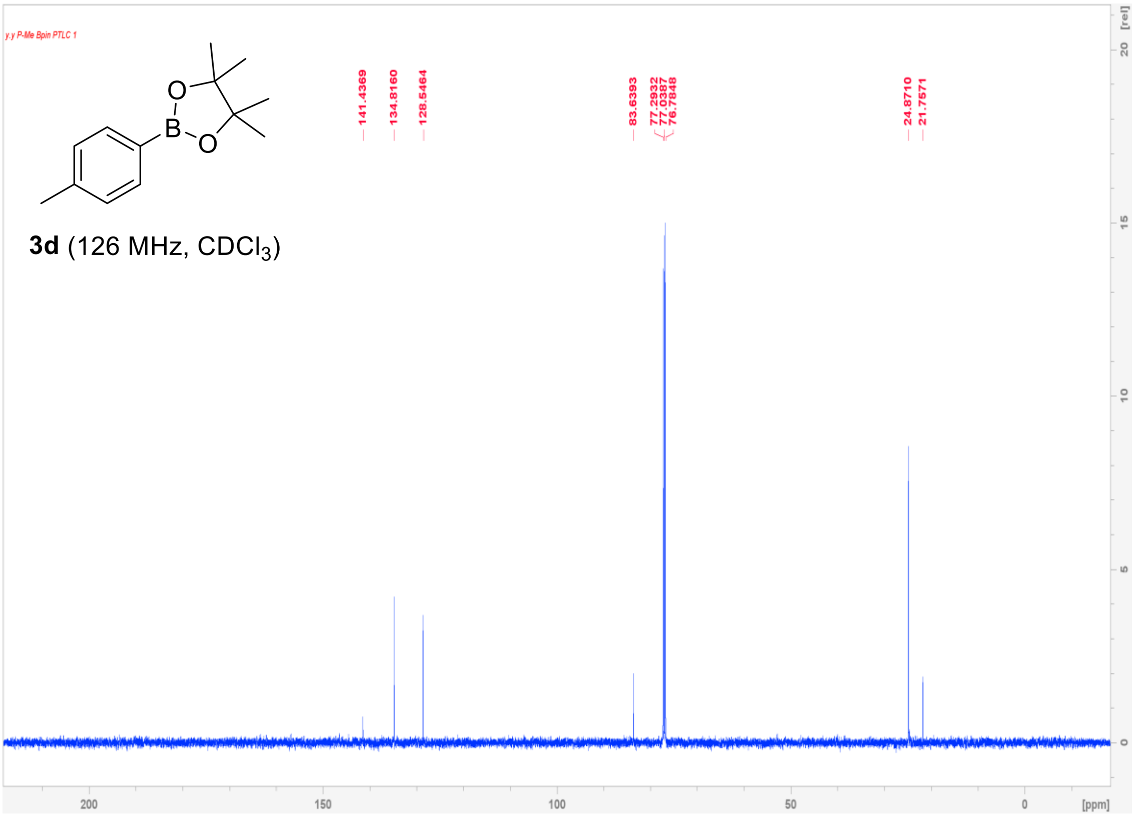

Figure S5. ^{1}H and ^{13}C{^{1}H} NMR spectra of compound **3e**

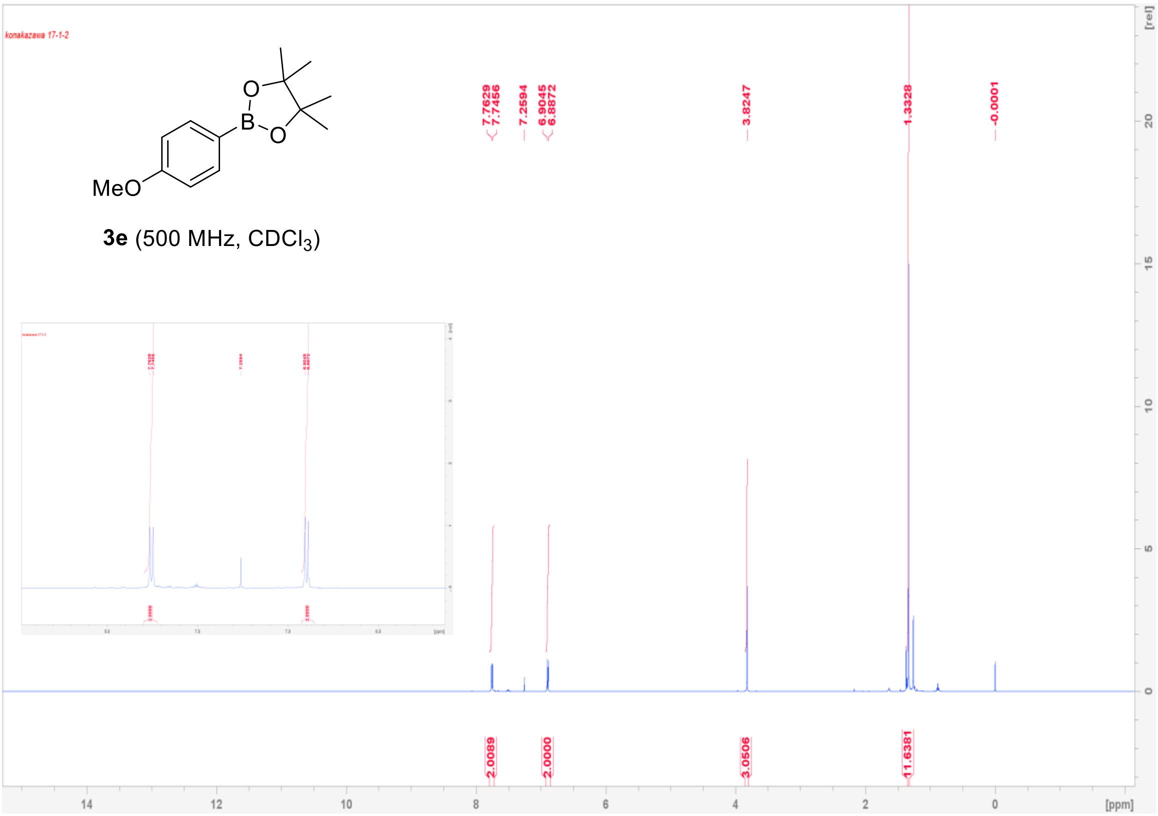

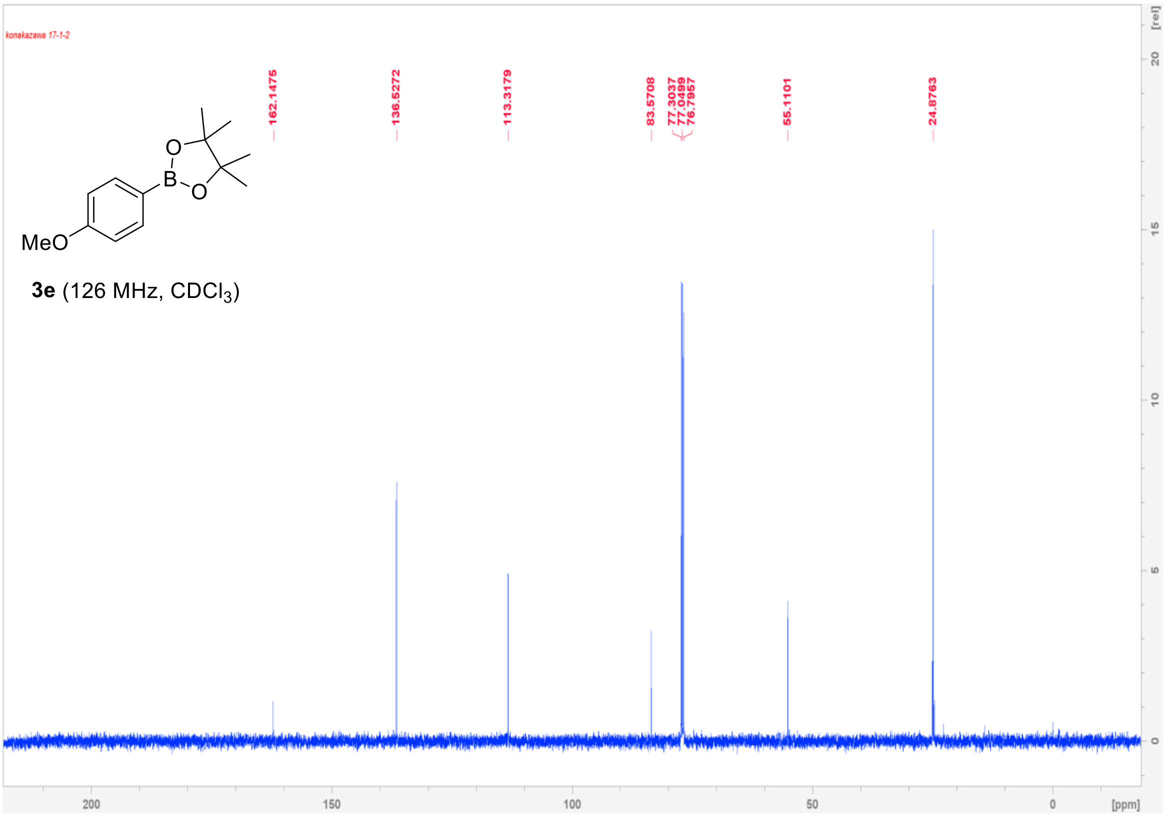

Figure S6. ^{1}H and $^{13}C\{^{1}H\}$ NMR spectra of compound **3f**

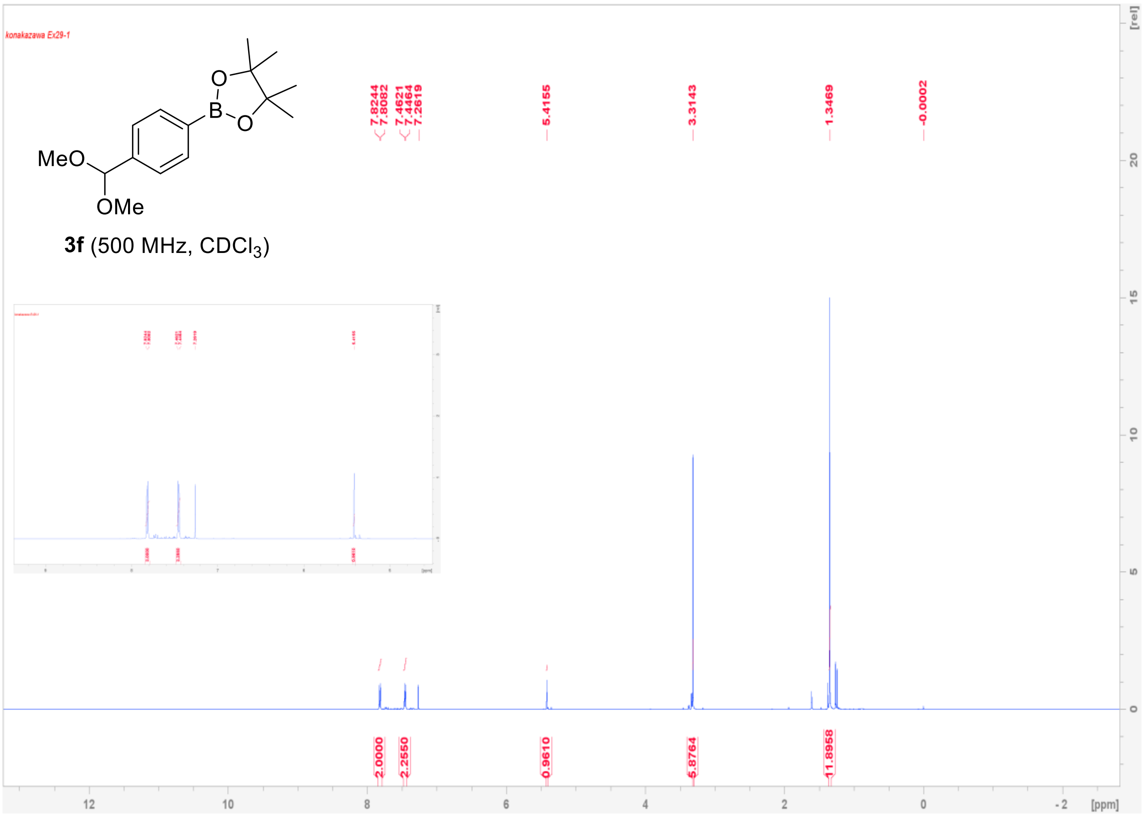

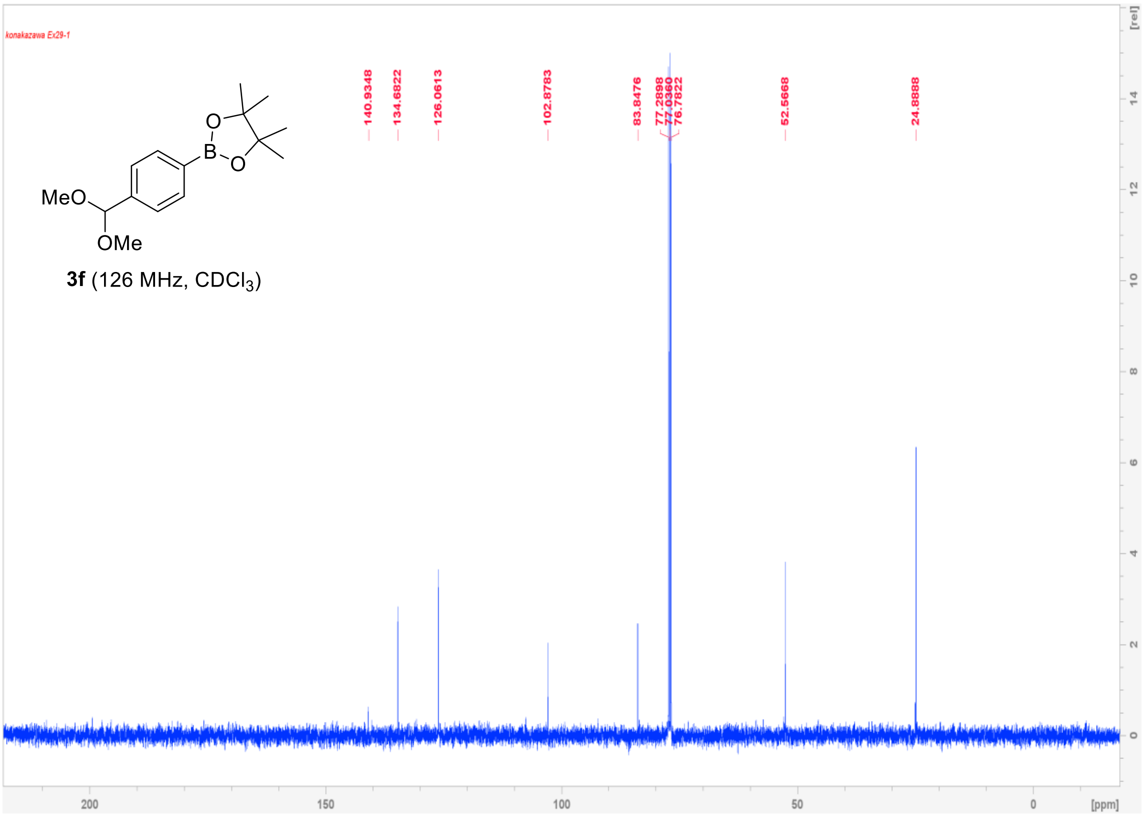

Figure S7. ^{1}H and $^{13}C\{^{1}H\}$ NMR spectra of compound **3g**

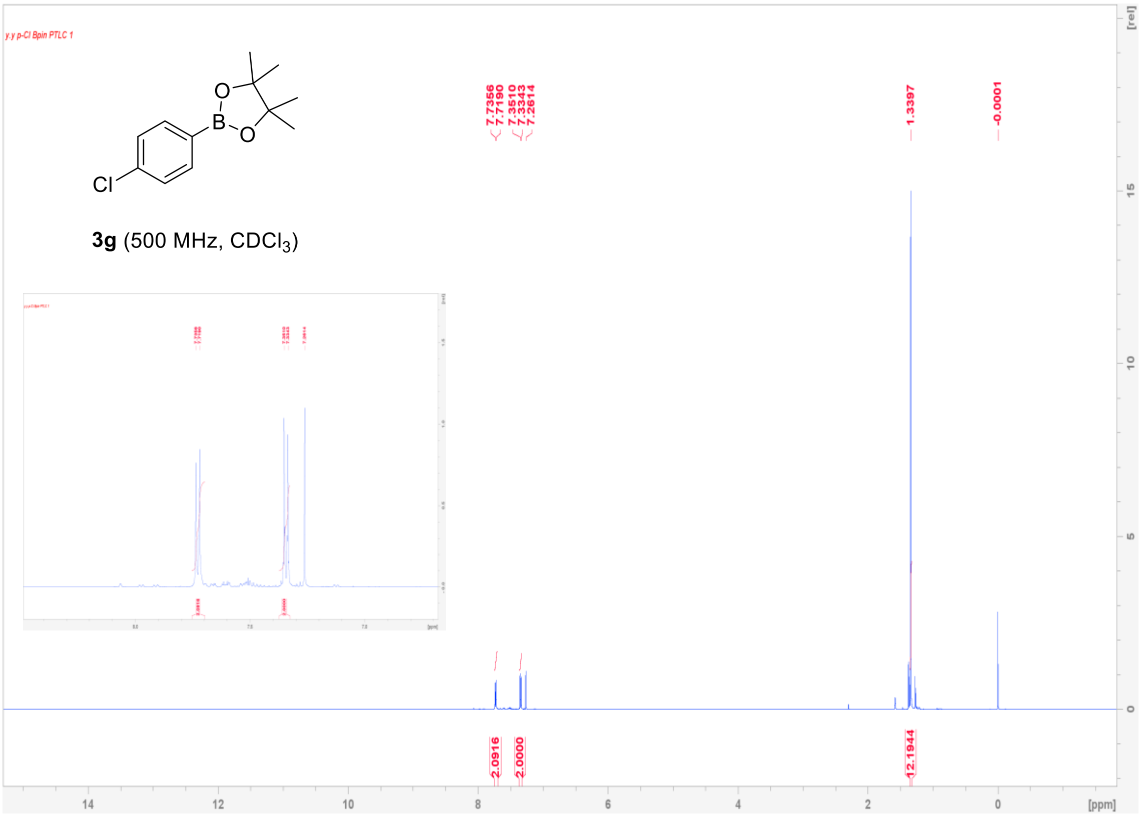

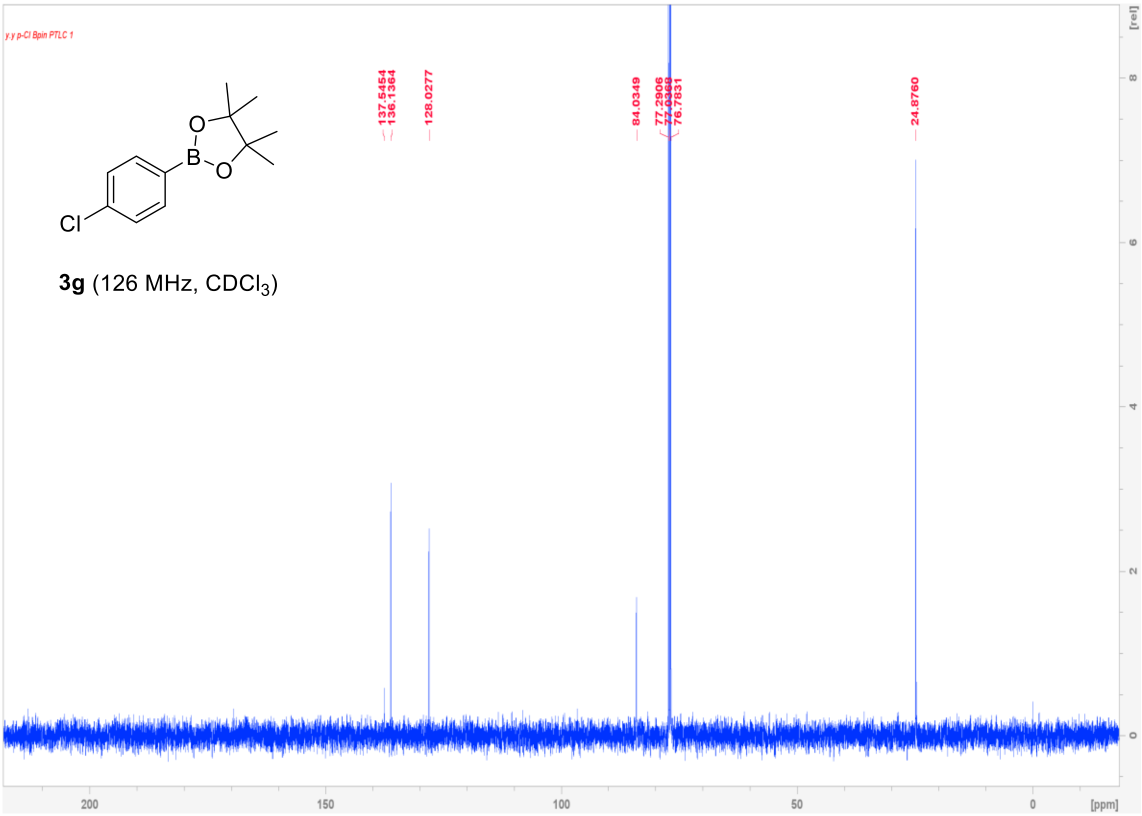

Figure S8. ^{1}H and $^{13}C\{^{1}H\}$ NMR spectra of compound **3l**

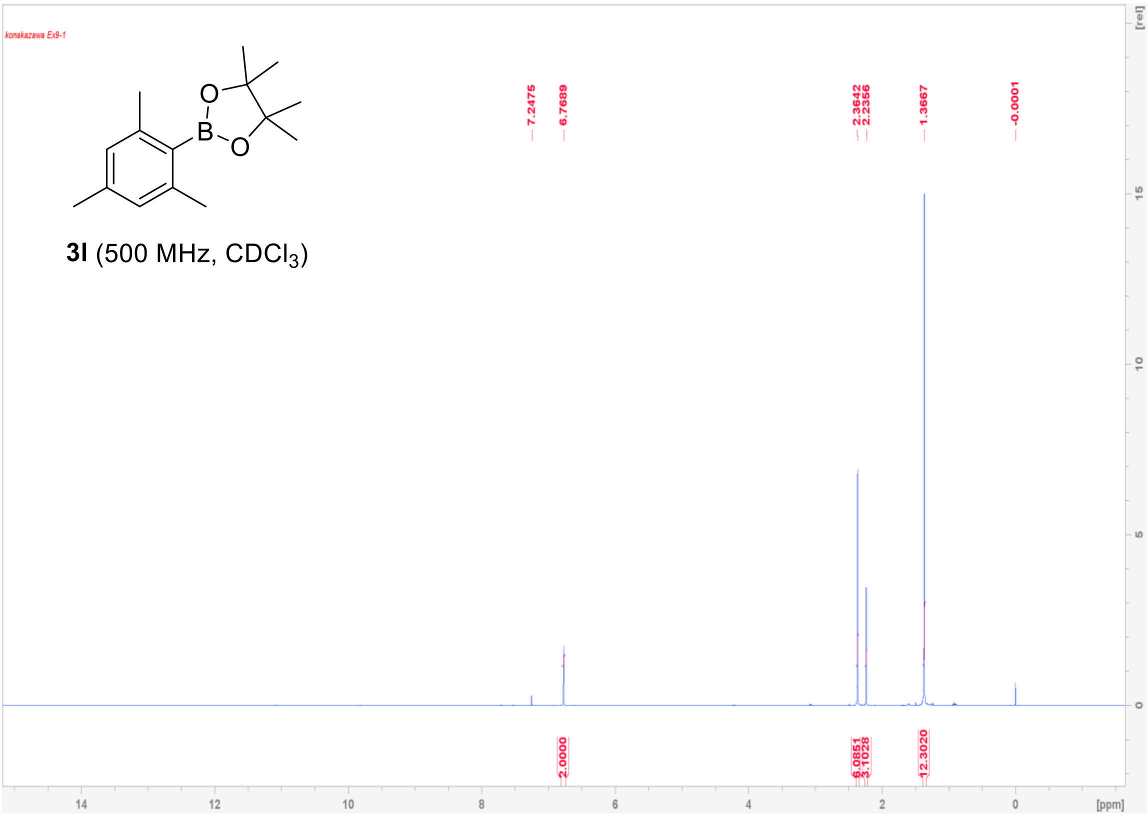

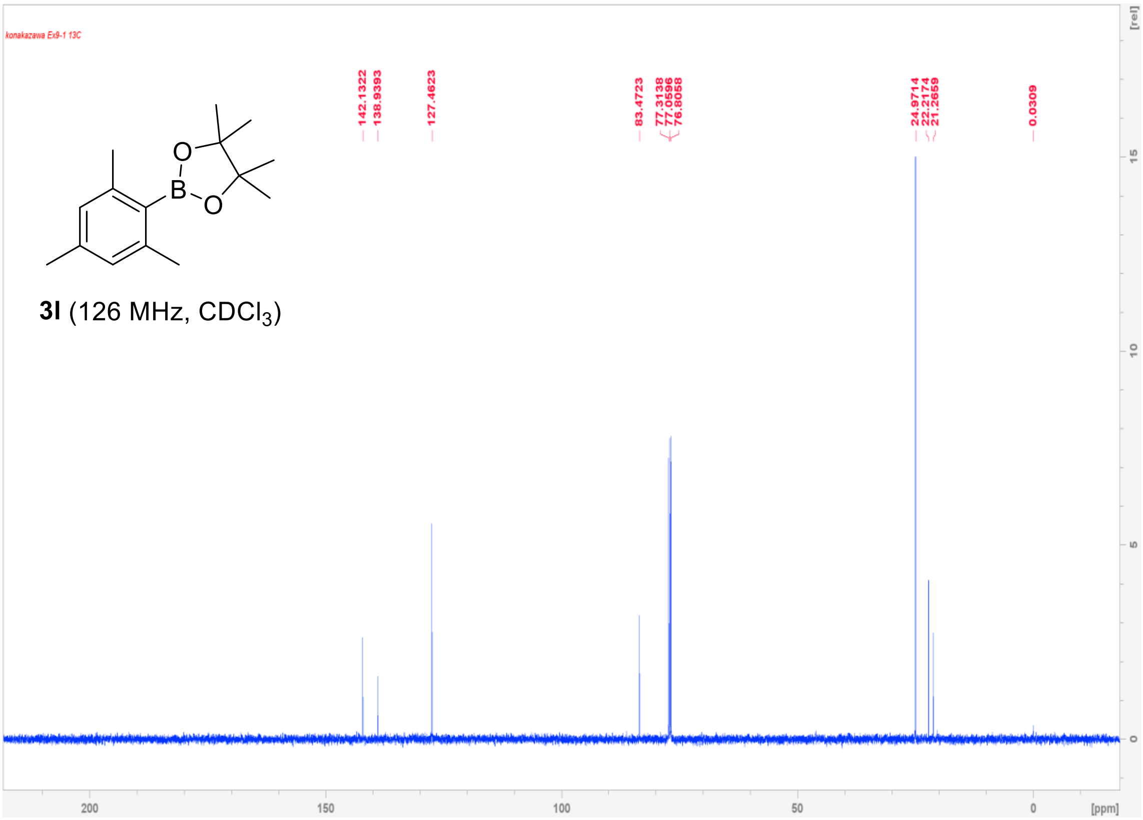

Supplement: File 1 — Investigation of the boron residue in the crude mixture by 11B NMR measurement, characterization data of the compounds, and copies of 1H NMR and 13C{1H} NMR spectra. [file Beilstein_J_Org_Chem-20-2577-s001.pdf]
